# Cystic tumor of the atrioventricular node

**DOI:** 10.21542/gcsp.2020.28

**Published:** 2020-11-30

**Authors:** Katherine Giuliano, Brandi Scully, Eric Etchill, Jennifer Lawton

**Affiliations:** 1 Division of Cardiac Surgery, Department of Surgery, Johns Hopkins University School of Medicine, Baltimore, Maryland, USA

## Abstract

We present a case of a 71-year-old female with complete heart block and an incidentally found atrioventricular nodal inclusion cyst.

## Case presentation

**Figure 1.**
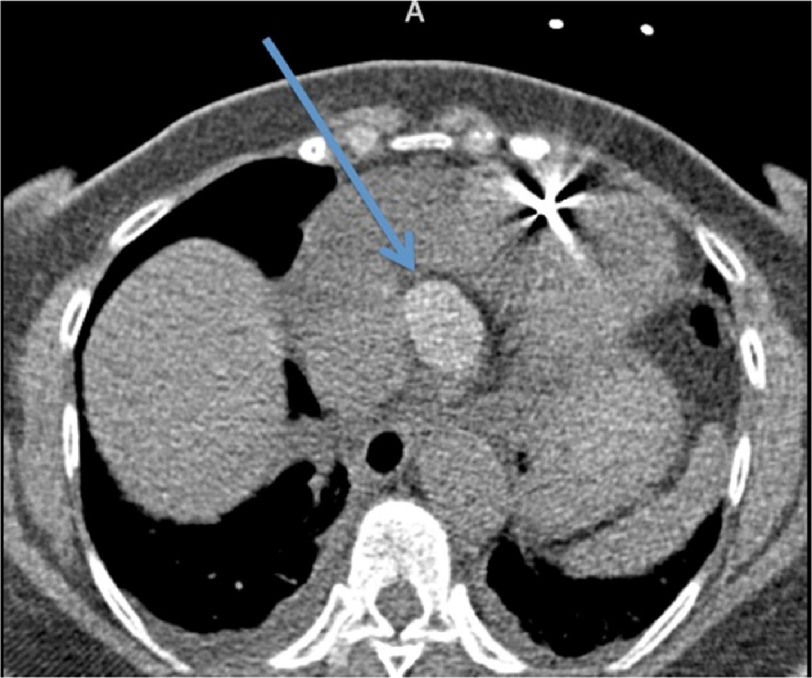
The mass was incidentally noted on an outpatient cardiac computerized tomography (CT) scan for calcium scoring. The arrow points to the 3.7 × 2.5 cm well-circumscribed, hyperdense mass on this axial cut.

**Figure 2.**
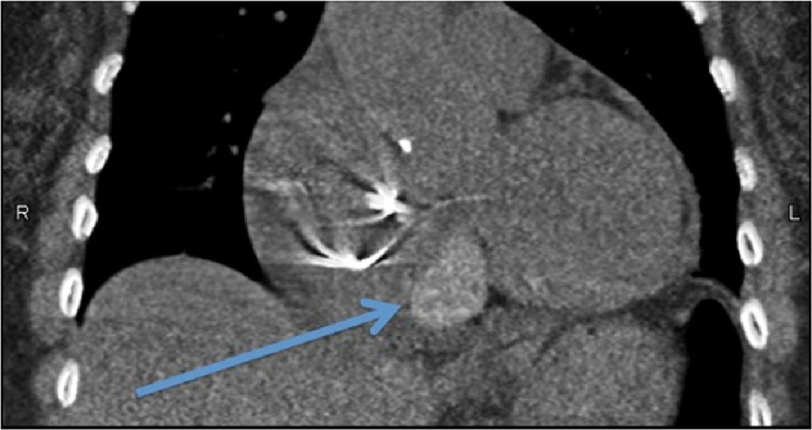
The mass was located at the anteroinferior aspect of the interatrial groove and to the right of the coronary sinus, as indicated by the arrow on this coronal cut of the patient’s cardiac CT for calcium scoring. Also visualized are her pacemaker leads.

The patient is a 71-year-old female with a past medical history of paroxysmal atrial fibrillation treated with apixaban, complete heart block status-post permanent pacemaker placement in 2008, hypertension, and congestive heart failure (CHF). She was admitted to the hospital for a CHF exacerbation after presenting with several weeks of lower extremity edema, fatigue, and a dry cough. Just prior to her hospitalization, she had undergone an outpatient computed tomography (CT) heart scan with a total calcium score of zero. This scan incidentally noted a 3.7 ×  2.5 cm well-circumscribed, hyperdense lesion at the anteroinferior aspect of the interatrial groove and to the right of the coronary sinus (Figures 1 and 2).

**Figure 3.**
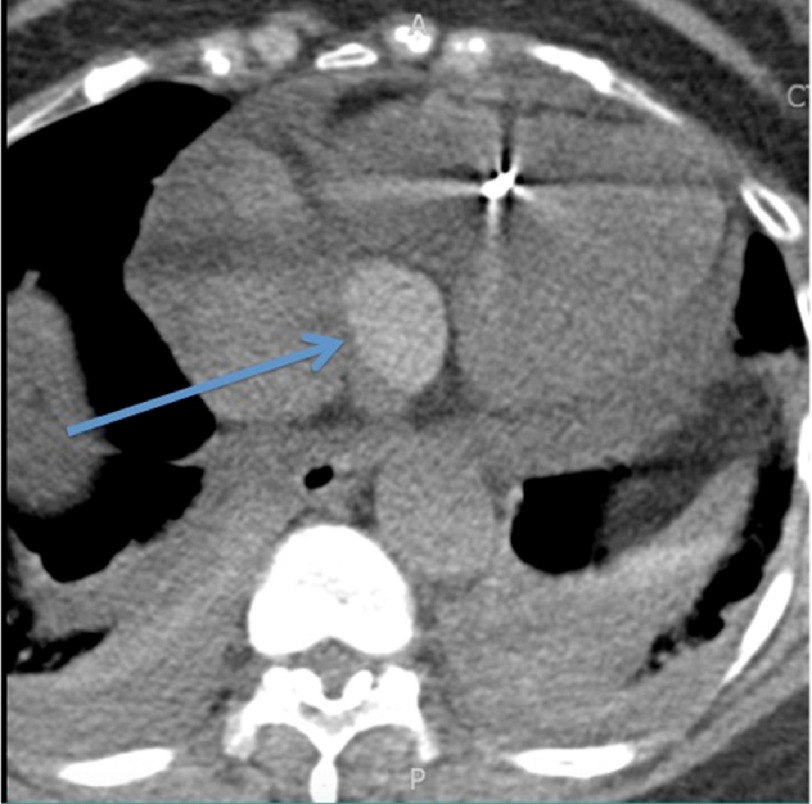
Magnified view of the hyperdense, ovoid, circumscribed structure along the inferior surface of the heart, as indicated by the arrow in this noncontrast axial slice of the CT angiogram (CTA).

**Figure 4.**
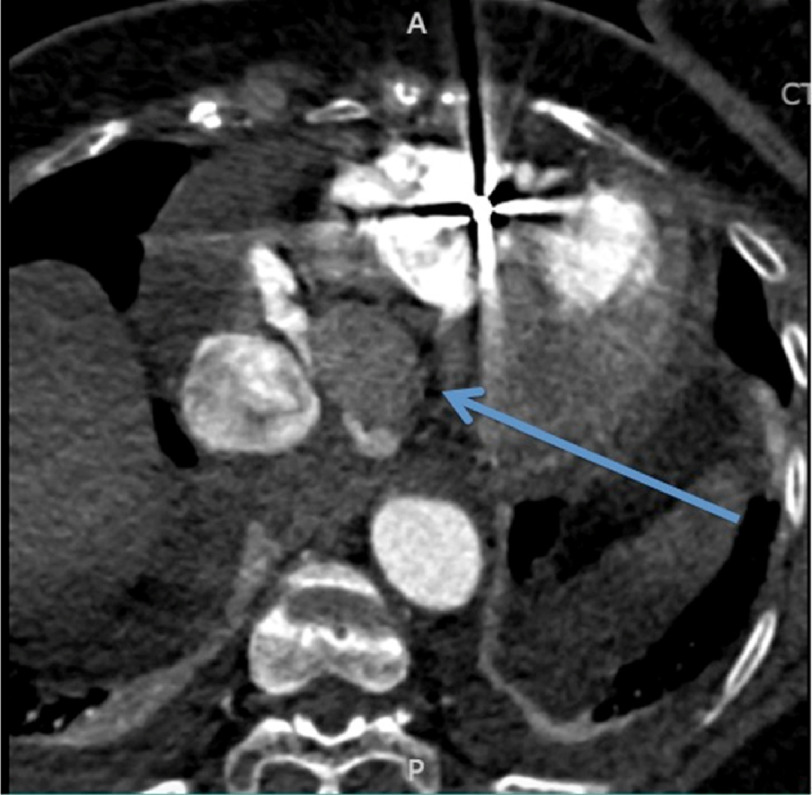
Axial slice showing a magnified view of the heart from a CTA of the chest with contrast, demonstrating (as indicated by the arrow) the mass with imaging features most suggestive of a cystic tumor of the atrioventicular (AV) node.

During her hospitalization, she underwent a CT angiogram (CTA) of the chest to further characterize this lesion. This demonstrated a hyperdense, ovoid, circumscribed structure along the inferior surface of the heart with imaging features most suggestive of a cystic tumor of the atrioventricular (AV) node (Figures 3 and 4). A CTA of the chest additionally showed cardiomegaly, a moderate-sized pericardial effusion, and small bilateral pleural effusions.

**Figure 5.**
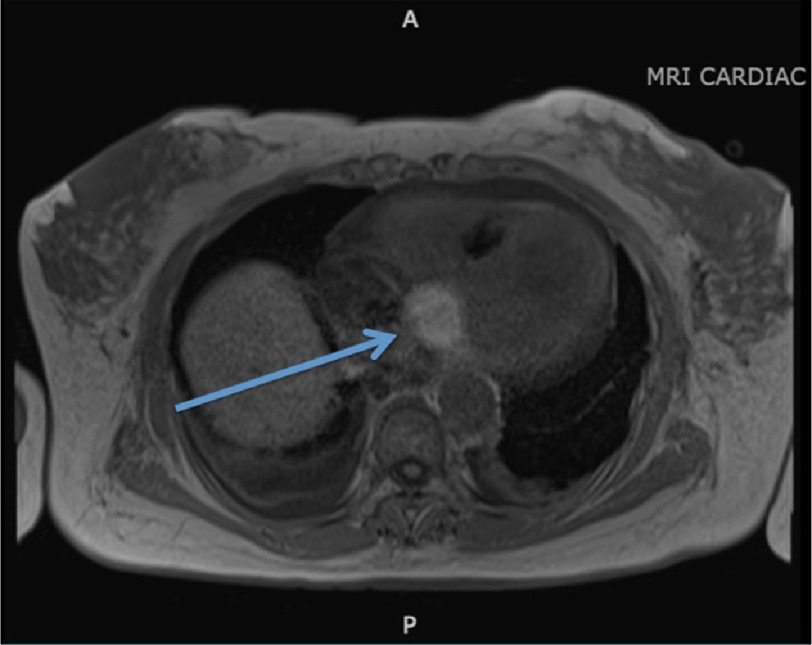
Axial slice of the patient’s cardiac magnetic resonance imaging (MRI). The arrow indicates the interatrial lesion centered within the region of the atrioventricular node.

**Figure 6.**
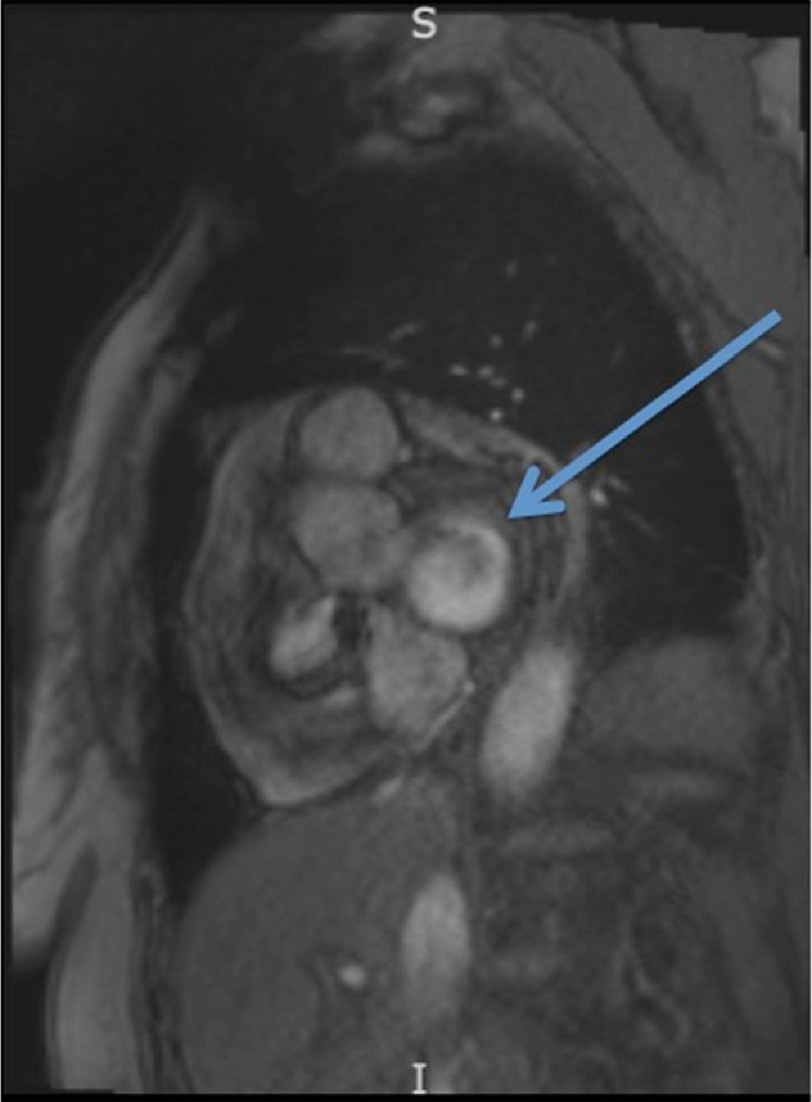
Sagittal view of the mass (indicated by the arrow) as seen on the patient’s cardiac MRI. Based on imaging findings, it was felt to be consistent with an atrioventricular node inclusion cyst.

The patient underwent cardiac magnetic resonance imaging (MRI), which showed findings compatible with hypertrophic cardiomyopathy as well as the interatrial lesion centered within the region of the AV node. This was interpreted to be consistent with an AV node inclusion cyst (Figures 5 and 6). She also underwent a transthoracic echocardiogram (TTE) that demonstrated severely reduced systolic function with a left ventricular ejection fraction (EF) estimated at 20–25%. This was in contrast to a normal EF (60–65%) on a TTE four months prior.

She underwent diagnostic left heart catheterization, which showed normal coronary arteries. She was medically managed for CHF exacerbation with diuresis and was discharged to home after a five-day hospitalization. Repeat TTE one month after discharge showed normal global left ventricular systolic function with an EF estimated at 55–60%. She was symptomatically improved and able to tolerate exercise three to four days per week without shortness of breath, chest pain, or lower extremity swelling. Cardiac surgery consultation was obtained, and observation and follow-up was recommended with repeat imaging in one year.

## Literature review

Primary cardiac tumors are uncommon. Cystic tumors of the AV node are especially rare, comprising an estimated 1–3% of all cardiac tumors.^1^ They occur in the area of the atrioventricular node along the atrial septum. They are composed of ectopic glands lined by cuboidal or squamous epithelium.^2^ While the tumors are considered benign, they often lead to conduction defects. Over 65% of patients have complete heart block, and another 15% of patients experience partial AV block.^3, 4^ Furthermore, cystic AV node tumors are the most common primary cardiac tumor causing sudden cardiac death.^2, 4^ Most commonly, however, these tumors are diagnosed postmortem incidentally at autopsy.^2, 5^ The tumors occur primarily in women, with a female-to-male ratio of 3:1, and at a mean age in the fourth decade.^2^

Given its rarity, the literature regarding this condition is from case reports only. As such, there is no standardized treatment plan, with surgical resection remaining controversial.^4, 5, 6^ The tumor itself is benign, but its associated risk of sudden cardiac death should be weighed against the risk of surgical resection.

## What have we learned?

Cystic tumors of the AV node are rare, comprising only 1–3% of all cardiac tumors. They are benign, but often lead to conduction defects and can cause sudden cardiac death. On CT scan, they appear as well-circumscribed, hyperdense lesions in the area of the AV node along the atrial septum. Given their rarity, there is no standardized treatment plan, with surgical resection remaining controversial.
